# Comparison of Penile Appearance and Outcomes Between Prefabricated Urethra and Pre-implanted Urethral Plate for Treatment of Children With Severe Hypospadias: A Retrospective Study

**DOI:** 10.3389/fped.2021.719551

**Published:** 2021-09-14

**Authors:** Yuan Ding, Shengli Gu, Xingrong Xia, Zhengbo Yu

**Affiliations:** Pediatric Surgery, The First People's Hospital of Zunyi (The Third Affiliated Hospital of Zunyi Medical University), Zunyi, China

**Keywords:** hypospadias, male urogenital abnormalities, staged surgery, retrospective study, penile

## Abstract

**Objective:** To compare the effect of prefabricated urethra and pre-implanted urethral plate in the treatment of severe hypospadias in children.

**Methods:** We retrospectively analyzed the clinical data of 53 patients who diagnosed as severe hypospadias underwent staging urethroplasty from January 2015 to January 2018 in the Department of Pediatric Surgery, First People's Hospital, Zunyi City. The patients were divided into two groups: group A (*n* = 25) were treated with prefabricated urethra and group B (*n* = 28) were treated with pre-implanted urethral plate. After the second stage surgery, the ratios of complications such as urethral fistula, urethral stenosis, urethrocele, and recurrence chordee were compared. The penis was scored from meatus, glans, shaft skin, general appearance by the parents, blinded urologists according to The Pediatric Penile Perception Score, and the scores were compared too.

**Results:** All patients were followed up after two stage operations for an average of 28 months. Glans dehiscence occurred in two patients (8%), urethral orifice stenosis occurred in one (4%) and urethral fistula occurred in three (12%) in group A. No urethral stenosis, urethrocele and recurrence chordee was observed. One patient presented urethral plate inactivation (3.6%), two patients presented urethral fistula (7.1%) and one patient presented urethral stenosis (3.6%) in group B. No urethrocele, glans dehiscence and recurrence chordee was observed. The total complication rate in group A was 24 and 14.3% in group B, respectively, and the difference was not statistically significant (*P* = 0.582). The differences between two groups scored by parents in glans (*P* = 0.030) was statistically significant. The differences between two groups scored by operators in meatus (*P* = 0.041), shaft skin (*P* = 0.000), glans (*P* = 0.001), and general appearance (*P* = 0.007) were statistically significant. The differences between two groups scored by counterparts in meatus (*P* = 0.006), shaft skin (*P* = 0.003), glans (*P* = 0.010), and general appearance (*P* = 0.014) were statistically significant.

**Conclusion:** Both prefabricated urethra and pre-implanted urethral plate methods are suitable for correction of severe hypospadias as staging surgery in children. In general, pre-implanted urethral plate is more worthy of spread because it is much more applied in patients with small glans and achieve good appearance of penis.

## Introduction

Hypospadias is one of the most common malformations of the pediatric genitourinary system. The incidence of hypospadias in male newborns is 1/250, and it has been gradually increasing in recent years (1). Hypospadias is corrected for cosmetic reasons, fertility, and to enable urinating when standing. Generally surgical repair is carried out at an early age and can significantly impact urinary and sexual function in adult life (2). Distal hypospadias is a more common occurrence in western countries, while proximal hypospadias cases are more commonly reported in Asia (3, 4). The etiology of hypospadias in the majority of cases remains unknown. Only a few risk factors have been found in association with hypospadias, for instance, paternal subfertility, intrauterine growth retardation, and low birth weight (5).

Due to serious deformity and long urethra defect in severe hypospadias, which make surgical treatment difficult, there is no unified surgical method for the treatment of hypospadias at present. Treatment is associated with a range of complications linked to urethroplasty, and commonly include fistula, obstruction to the neourethra (by meatal stenosis or urethral stricture), or wound dehiscence (6). Although many treatment approaches for hypospadias exist, the two main treatment methods are one-stage and multi-stage formation. However, due to the complications involved in single-stage surgery, as high as 21–51% (7, 8), many surgeons prefer staging surgery. In some cases, the second stage of urethroplasty can pose a challenge due to graft shrinkage, meatal stenosis, or small glans (9). Identifying and taking the ideal surgical approach to treat hypospadias is vital because surgeries that previously failed have been reported to result in tissue ischemia because of hypo vascularization, reduced perfusion pressure, and scarred penile tissue at the previous suture line, and any further attempts to correct a previously failed surgery have been linked to a high failure rate ranging from 14–56% (10, 11).

Some studies indicated that the outcomes of single and multistage repair of proximal hypospadias are comparable (12). But Ramesh concluded from his meta-analysis that two-stage repair of proximal hypospadias had significantly less complications compared to single stage repair (*p* = 0.01) (13). Compared to one-stage surgery, staging surgeries can distribute the difficulty and risks of the surgery to different stages, the postoperative appearance is more satisfactory, and there is a lower rate of complications. At present, more and more medical professionals are opting for staging surgeries to treat severe hypospadias (11, 14–16). Despite the general preference for staging surgeries and the reported successes with staging repair, about 40% of men treated for severe hypospadias have voiding problems to some degree, and over 20% of men reported having sexual problems (17). However, data of short-term and long-term outcomes of severe hypospadias repair are limited.

Few researchers have comparatively analyzed two different types of staging surgery for treating severe hypospadias (18). Subramaniam et al. stated that there are as many reconstructive techniques and modifications for hypospadias repair as there are surgeons performing it (8). To our knowledge, there are no other comparative studies on different types of staging surgeries for treating hypospadias in China. As it still early to come to a consensus based on outcomes and provide appropriate guidance, performing a comparison of multi-stage surgeries will help provide a better picture of the most appropriate surgical approach for the repair of hypospadias in children.

Therefore, in the present study, we retrospectively analyzed the clinical data of patients who underwent staging surgery for severe hypospadias using prefabricated urethra and pre-implanted urethral plate and we compared the postoperative effect of these two types of surgeries in the short term.

## Materials and Methods

### Research Design and Research Subject

This retrospective study included individual clinical data of patients with severe hypospadias who had been treated in the Department of Pediatric Surgery, First People's Hospital of Zunyi City from January 2015 to January 2018. All patients with proximal hypospadias were included in the analysis provided they met the following criteria: the urethral opening was located in the penile sac, scrotum, and perineum after the penis was straightened during the surgery. Cases were diagnosed as proximal hypospadias when, after degloving the penis, the meatal location was at or proximal to the penoscrotal junction (19). Patients who met one or more of the following exclusion criteria were excluded from the analysis: urethral opening located on the body of the penis, coronal sulcus, or glans penis, as well as patients who have previously undergone one-stage surgery in another hospital.

**Figure 1:**
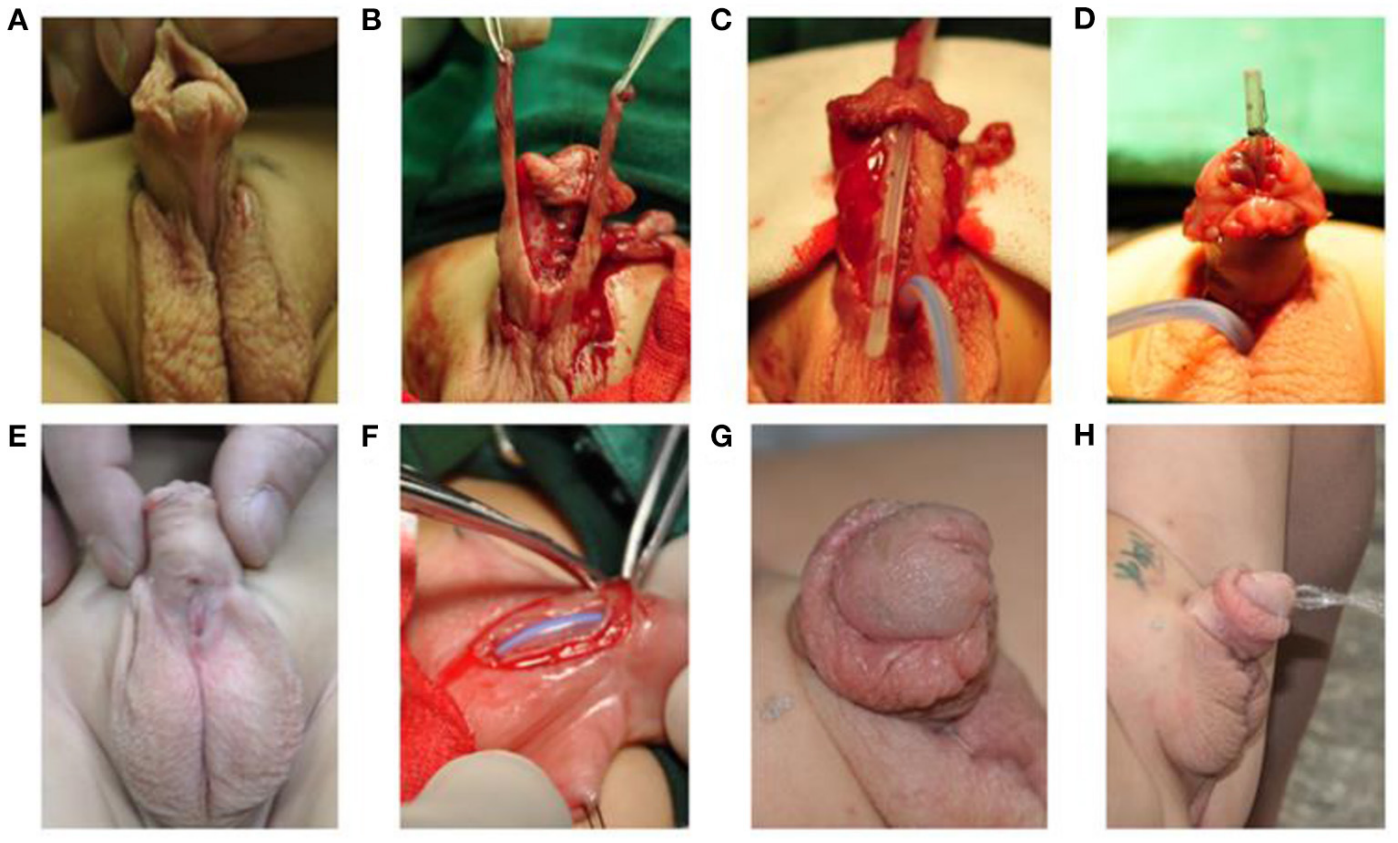
Schematic plot for prefabricated urethra staged surgery. **(A)** Appearance before the first stage surgery; **(B)** “Wing-shaped” skin flaps separated from around the urethra; **(C)** Urethra formed by rolling the skin flap; **(D)** Support tube retained in the prefabricated urethra; **(E)** appearance before the second-stage surgery; **(F)** Urethra formed by anastomosis of the skin flap around the freed fistula; **(G)** Appearance after the second stage surgery; **(H)** Urination after the second-stage surgery.

**Figure 2:**
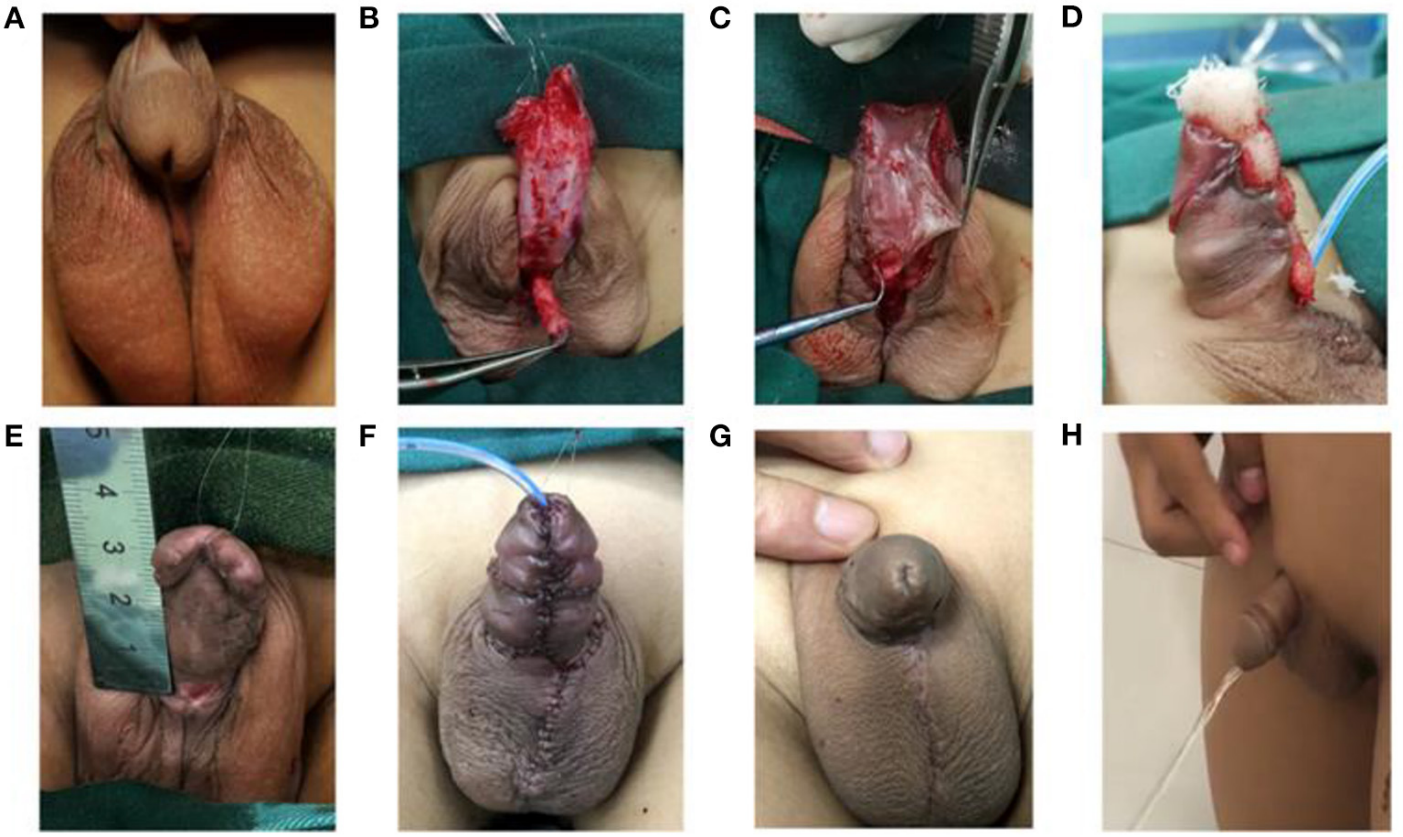
Schematic plot for pre-implanted urethral plate staged surgery. **(A)** Appearance before the first-stage surgery; **(B)** The urethra is freed and the penis is straightened; **(C)** The freed skin flap is sutured at multiple points for fixation; **(D)** Vaseline gauze is used between the sutures; **(E)** Appearance before the second stage of the surgery; **(F)** Appearance after sutures in the second stage of surgery; **(G)** Appearance after the second surgery; **(H)** Urination after the second surgery.

The two different surgical procedures were the prefabricated urethral staging surgeries and the pre-implanted urethral plate staging surgeries. Patients were explained surgical procedures in detail, and the final decision as to which surgical procedure to accept was determined by the doctor and the patient together. All the patients signed informed consents for the surgical procedure. All surgeries were performed by the same surgeon, i.e., the chief physician who has worked in this profession for more than 30 years. All the patients who were receiving one of the staging surgeries, were finally grouped into the prefabricated urethral staging surgeries (group A) and pre-implanted urethral plate staging surgeries (Group B). The patients were selected according to the morphological characteristics of the prepuce, including the resilience, smoothness and available area. Once the prepuce was favorable and enough to be tubularized, the patient was assigned to group A (Figure 1A), if not, the patient was assigned to group B (Figure 2A). This study was approved by the Ethics Committee of First People's Hospital of Zunyi City (Approval No: 2019-103). The requirement to obtain informed consent for this study was waived by the Ethics Committee due to its retrospective nature.

### Staging Surgery Procedure for Prefabricated Urethra

Surgical repair of severe hypospadias using prefabricated urethra was performed in two stages (20). The first stage involved penile straightening and prefabrication of the urethra. The surgeon made a circular incision on the foreskin 0.5 cm from the coronal sulcus, transected the urethral plate, detached the penis, reached deep into the Buck's fascia, and completely loosened and removed the scar tissue on the ventral side of the penis. Then, three parallel transverse cuts were made on the ventral side tunica albuginea of the cavernous body of the penis (three transverse corporotomies) to extend and straighten the penis, and a longitudinal incision was made along the penile skin at 0.8 cm on either side of the urethral opening, separating two vascularized “wing-like” flaps to the glans (Figure 1B). The length of each flap is the actual length from the original urethral opening to the glans after straightening. Subsequently, the vascularized flaps were transferred to the distal end of the prefabricated urethra formed by ventrally sutured roll tube, establishing a glans tunnel. Then, the distal neo-urethra was sutured and fixed to the glans (Figure 1C). The vascularized tissue of the flap was sutured on the top and the distal end of the prefabricated urethra was sutured to form the glans. A fistula was placed between the original urethral opening and the proximal end of the prefabricated urethra. The foreskin flap was designed and sutured. An F6-8 foley catheter was inserted from the original urethral opening into the bladder, and a piece of F6 silicone support tube was inserted into the prefabricated urethra (Figure 1D). After the surgery, the patients were administered antibiotics for 24 h as prophylaxis. The foley catheter was retained for 10 days, and the support tube was retained for 7 days.

The second stage of the surgery involved anastomosis of the original urethral opening and the proximal opening of the prefabricated urethra (Figure 1E). This was performed 6 months after the first stage (Figure 2E). The surgeon inserted an F6 foley catheter into the urethral opening in the glans, which entered the bladder through the anastomosis; made a circular incision at 0.4 cm around the original urethral opening and the proximal opening of the prefabricated urethra (Figure 1F); separated the flaps and flipped the flap to perform an anastomosis; took one side of the pedicled periorchium to strengthen the covering suture; and finally designed and sutured the foreskin flap. Postoperatively, antibiotics were used for 24 h as prophylaxis, and the catheter was retained for 7 days.

### Staging Surgery Procedure for Preimplanted Urethral Plate

Two-stage repair was performed to treat severe hypospadias with a pre-implanted urethral plate (21). The first stage of the surgery involved penile straightening and pre-implanted the urethral plate. The surgeon made a circular incision on the foreskin at 0.5 cm from the coronal sulcus, transected the urethral plate, detached the penis, reached deep into Buck's fascia, completely loosened and removed the scar tissue on the ventral side of the penis, freed the urethra from the bulbospongiosus (Figure 2B). Then, three parallel transverse cuts were performed on tunica albuginea on the ventral side of the cavernous body (three transverse corporotomies) to extend and straighten the penis. The freed urethra was sutured and fixed in the middle of the penis on the ventral side in a naturally straight state. An incision was made on the ventral side of the glans penis through the middle, and the two wings of the glans were fully freed and unfolded to 180°. The inner plate of the foreskin was cut to a length equal to the actual length from the original urethral opening to the glans after the penis is straightened and the width was about 2.0 cm. The foreskin was cut longitudinally in the middle of the penis dorsally, flipped to the ventral side and then sutured. The free skin flaps, from which the hypodermis had been removed, was sutured to the ventral cavernous body of the penis intermittently at multiple points (Figure 2C). Then, an F6-8 foley catheter was inserted from the original urethral opening into the bladder, and Vaseline gauze rolls were placed on the ventral side of the penis and sutured for fixation (Figure 2D). Postoperatively, antibiotics were used for 24 h as prophylaxis, the gauze was removed on day 8, and the catheter was retained for 10 days.

The second stage of the surgery (urethroplasty) was performed 6 months after the first stage. A longitudinal incision ~2.0 cm wide was made on the urethral plate transplanted from the glans and the ventral side of the penis. A “U”-shaped incision was made from the proximal end bypassing the urethral opening. The skin and subcutaneous tissue on both sides of the urethral plate were loosened, and both wings of the glans were fully unfolded. An F6 foley urinary catheter was then inserted into the bladder, and the urethra opening was continuously introverted and sutured to form a urethra orifice on the glans. One side of the pedicled periorchium was used to strengthen the covering suture, and the two wings of the glans were sutured to construct the glans penis. Then, the foreskin flap was designed and sutured (Figure 2F). Postoperatively, antibiotics were used for 24 h as prophylaxis, and the catheter was retained for 7 days.

### Data Collection, Follow-Up, and Outcomes

After the second stage of the surgery, follow-up visits were made in January, March, June, and December every year for 5 years, mainly to observe the appearance of the penis, and complications such as urinary fistula, urethrostenosis, urethrocele, and recurrence chordee. The total incidence of complications determined during follow-up visits were considered the study's outcome. We used the same method as Daniel et al. to make PPPS (pediatric penile perception score). The specific scoring items include configuration and position of the meatus, configuration and appearance of the glans, appearance of the shaft skin and general appearance. Three months after operation, patients or their parents, operators and counterparts could express their satisfaction for every single item according to a 4-point Likert scale, which included the ratings of very dissatisfied (0 points), dissatisfied (1), satisfied (2) and very satisfied (3). The PPPS was calculated by adding the scores of the item's meatus, glans, shaft skin and general appearance.

### Statistical Analysis

Data were analyzed using Statistical Package for Social Sciences (SPSS) (version 25.0; IBM, Chicago, IL). The Kolmogorov-Smirnov (KS) normality test was performed to estimate distribution of the measurement data. Data that conformed to a normal distribution were expressed as means ± standard deviation, and independent sample *t* test was used to compare differences between groups. Data that did not conform to a normal distribution were expressed as M (P_25_-P_75_), and Wilcoxon rank-sum test was used to compare differences between groups. Count data were expressed as *n* (%), and the chi-square (χ^2^) test or Fisher's exact test was used to compare the incidence of complications between the two groups. A *P* value of <0.05 was used to denote statistical significance.

## Results

### Clinical Features

A total of 53 cases were included in this retrospective study. The subjects' ages ranged from 1 year 3 months to 8 years 2 months (average, 2 years 6 months). There was no statistical significance in the comparison of the clinical measurement data in the two groups, such as differences in the age, glans width, width of the urethral plates, width of the navicular groove and the length of the defected urethra (*P* > 0.05; Table 1). The study subjects had the following types of hypospadias: penile-scrotal type (*n* = 31; 15 and 16 in groups A and B, respectively), scrotal type (*n* = 13; 6 and 7 in groups A and B, respectively), and perineal type (*n* = 9; 4 and 5 in groups A and B, respectively). Of the 53 cases, 25 underwent prefabricated urethral staging surgery (group A) and 28 underwent pre-implanted urethral plate surgery (group B). All the patients underwent an artificial erection test to measure the angle of chordee with a protractor. In group A, the angle was moderate (30°-35°) in 5 cases and severe (>35°) in 20; and in group B, the angle of chordee was moderate (30°-35°) in 7 and severe (>35°) in 21. All the patients underwent chromosomal examination before surgery to ensure they had a chromosomal karyotype of 46XY. In group A, two cases of the perineal type also had unilateral cryptorchidism. Disorders of Sex Development (DSD) were excluded by preoperative abdominal gonadal ultrasound and inguinal ultrasound and intraoperative exploration. Baseline patient data showed no significant differences between the two groups (*P* > 0.05; Table 2).

**Table 1:** Comparison of clinical measurement data between two groups.

**Clinical features**	**Group A**, ***n*****= 25**	**Group B,** ***n*** **= 28**	**Z/t**	***P***
Age (months)	29 (21, 37)	31.5 (21, 40.25)	−0.357	0.721^a^
Width of the glans (mm)	13.88 ± 1.58	13.70 ± 1.56	0.415	0.68^b^
Width of the urethral plate (mm)	5.12 ± 0.87	5.07 ± 1.01	0.202	0.841^b^
Width of the navicular groove (mm)	2.70 ± 0.51	2.70 ± 0.60	−0.023	0.982^b^
Length of defective urethra (mm)	40.50 (33.65, 42.55)	38.55 (32.80, 42.5)	−0.455	0.649^b^

*Group A: prefabricated urethral staging surgeries; Group B: pre-implanted urethral plate staging surgeries*.

*All data displayed as means ± standard deviation or median and inter-quartile range*.

a *Represents Wilcoxon (Z) rank-sum test, and*

b *represents the t test*.

**Table 2:** Comparison of the clinical diagnostic type and degree of chordee between the two groups.

**Clinical features**		**Group A,** ***n*** **= 25**	**Group B,** ***n*** **= 28**	***X*** ^**2**^	***P***
Diagnostic type, *n* (%)	Penile-scrotal type	15 (60.0)	16 (57.1)	0.143	>0.999^c^
	Penile-scrotal type	6 (24.0)	7 (25.0)		
	Perineal type	4 (16.0)	5 (17.9)		
Degree of chordee, *n* (%)	Moderate	5 (20.0)	7 (25.0)	0.189	0.664
	Severe	20 (80.0)	21 (75.0)		

*Group A: prefabricated urethral staging surgeries; Group B: pre-implanted urethral plate staging surgeries*.

c *Represents Fisher's exact test*.

### Postoperative Follow-Up for Prefabricated Urethra

The postoperative follow-up period of our patient ranged from 12–60 (22) months. The urethroplasty at the glans in group A was divided into two types: ventral incision embedding method and tunnel method. After the first stage of the surgery using the ventral incision embedding method, there were two cases (8%) of glans dehiscence. After the first-stage surgery using the tunnel method, there was one case (4%) of external urethral orifice stenosis, and three cases where the glans diameter was <1.4 cm. After treatment with human chorionic gonadotropin (hCG), the glans was reshaped and recovered during the second stage of surgery. There were two cases of urinary fistula. In this group, there were two cases of perineal type with unilateral cryptorchidism and one case had bilateral cryptorchidism. The total rate of complications for both stages was 24%. For some patients, the penis appeared bloated, and although the urethral opening was located in the front of the glans, void with a good streamit, it did not form a longitudinal fissure (Figures 1G,H).

### Postoperative Follow Up for Preimplanted Urethral Plate

In group B, after the first-stage surgery, there was one case (3.6%) with severe chordee suffered from partial infection and inactivation of the pre-implanted urethral plate. During surgery, the ventral side tunica albuginea of the cavernous body of the penis was cut open and the penis was straightened. After irrigation with local injection of epinephrine saline (1:200,000 dilution) and bipolar electrocoagulation hemostasis, no obvious bleeding was seen. During unpacking, the inner dressing was found to be soaked with blood, which was considered to be the hematocele between the freed skin flap and the cavernous body of the penis. The patient recovered following hyperbaric oxygen therapy and changing of the local dressing. After second-stage surgery, there were two cases (7.1%) of urinary fistula, which was cured after another surgery. There was one case of urethrostenosis (3.6%), which was cured after urethral dilatation and retention of F8 foley urethral catheter for 1 month. The penis did not appear bloated, the urethral opening was in the front of the glans, void with a good streamit and appeared as a longitudinal fissure (Figures 2G,H).

There were two cases (7.1%) of urinary fistula in the prefabricated urethral group, which were both repaired in the second stage of the surgery. There was one case (4%) of inactivation of the urethral plate after the second stage of the surgery, which was repaired by another surgery. There was one case of urethrostenosis (3.6%). There were no complications such as urethrocele, external urethral orifice stenosis, or recurrence of chordee. The total complication rate of group B was 14.3%. The rates of complications did not significantly different between two groups (*P* = 0.582; Table 3).

**Table 3:** Postoperative complications between the two groups.

**Complications**	**Group A,** ***n*** **= 25**	**Group B,** ***n*** **= 28**	***X*** ^**2**^	***P***
Inactivation of the urethral plate	–	1 (3.6)	-	-
Urinary fistula	3 (12)	2 (7.1)	0.018	0.894
Dehiscence of the glans penis	2 (8)	0 (0)		0.218^c^
External urethral orifice stenosis	1 (4)	0 (0)	-	0.472^c^
Urethrostenosis	0 (0)	1 (3.6)	-	>0.999^c^
Urethrocele	0 (0)	0 (0)	-	-
Recurrence of chordee	0 (0)	0 (0)	-	-
Total number of complications	6 (24)	4 (14.3)	0.303	0.582

*Group A: prefabricated urethral staging surgeries; Group B: pre-implanted urethral plate staging surgeries*.

c *Represents Fisher's exact test*.

### Comparison of PPPS

The differences between two groups scored by parents in glans (*P* = 0.030) was statistically significant. The difference in shaft skin (0.396), meatus (0.062), and general appearance (0.069) were not statistically significant (Table 4). The differences between two groups scored by operators in meatus (*P* = 0.041), shaft skin (*P* = 0.000), glans (*P* = 0.001) and general appearance (*P* = 0.007) were statistically significant (Table 5). The differences between two groups scored by counterparts in meatus (*P* = 0.006), shaft skin (*P* = 0.003), glans (*P* = 0.010), and general appearance (*P* = 0.014) were statistically significant (Table 6).

**Table 4:** Comparison of two groups in PPPS by parents 3 months after operation.

**Clinical features**	**Group A,** ***n*** **= 25**	**Group B,** ***n*** **= 28**	***Z***	***P***
Meatus	2 (2, 3)	2 (2.5, 3)	−1.864	0.062
Glans	2 (2, 2.5)	2.5 (2, 3)	−2.165	0.030
Shaft skin	2 (2, 2.5)	2 (2, 3)	−0.848	0.396
General appearance	2 (2, 2)	2 (2, 3)	−1.820	0.069

*Group A: prefabricated urethral staging surgeries; Group B: pre-implanted urethral plate staging surgeries*.

**Table 5:** Comparison of two groups in PPPS by operators 3 months after operation.

**Clinical features**	**Group A,** ***n*** **= 25**	**Group B,** ***n*** **= 28**	***Z***	***P***
Meatus	2 (2, 2)	2 (2, 3)	−2.040	0.041
Glans	2 (2, 2)	2 (2, 3)	−3.660	0.001
Shaft skin	2 (2, 2)	2 (2, 3)	−3.182	0.000
General appearance	2 (2, 2)	2 (2, 3)	−2.711	0.007

*Group A: prefabricated urethral staging surgeries; Group B: pre-implanted urethral plate staging surgeries*.

**Table 6:** Comparison of two groups in PPPS by counterparts 3 months after operation.

**Clinical features**	**Group A,** ***n*** **= 25**	**Group B,** ***n*** **= 28**	***Z***	***P***
Meatus	2 (2,2)	2 (2, 3)	−2.750	0.006
Glans	2 (2,2)	3 (2, 3)	−2.582	0.010
Shaft skin	2 (2, 2)	2 (2, 3)	−2.995	0.003
General appearance	2 (2, 2)	2 (2, 3)	−2.461	0.014

*Group A: prefabricated urethral staging surgeries; Group B: pre-implanted urethral plate staging surgeries*.

## Discussion

In the present retrospective study, we compared the effect of prefabricated urethra and pre-implanted urethral plate in treating severe hypospadias. Among the 53 patients included, 25 and 28 patients underwent two-stage surgery with prefabricated urethra and pre-implanted urethral plate, respectively, and followed up for an average of 28 months. The results revealed that pre-implanted urethral plate repair led to a more satisfactory appearance.

More than 300 approaches exist to treat hypospadias both within China and internationally; however, none of these are considered as the best surgical method by all physicians (23). The Glans-Urethral Meatus-Shaft (GMS) hypospadias score could provide guidance for the selection of surgical methods to treat severe hypospadias, in which the presence and the severity of the chordee are the key factors in selecting the surgical method (24). Severe hypospadias often occurs together with moderate or severe chordee. The difficulty of surgery and the incidence of postoperative complications are relatively high. Therefore, the surgical method for severe hypospadias is worthy of more attention and discussion. Currently, there is no consensus on whether severe hypospadias should be surgically treated in one stage or multiple stages. Whatever the method used, it should reach the recognized standard of cure (25) as outlined below: (1). complete correction of chordee; (2). the urethral orifice is located at the head of the penis; (3). the appearance of the penis is satisfactory, and the individual can urinate in a standing position, and adult individuals are capable of normal sexual function. Currently, the curative effect of hypospadias is not limited to the elimination of complications such as urinary fistula, urethrostenosis, and urethrocele. In addition to eliminating these complications, the urethral orifice should present as a normal longitudinal fissure after circumcision, and the patient should have no psychological barrier after being an adult, which is the most essential goal of the treatment (26). Several studies have reported the complication rates of single- and multiple-stage surgery to repair severe hypospadias. Staging surgery is in fact a simplification of the difficult steps involved in the repair to reduce the occurrence of complications. Therefore, now it is advocated that one-stage surgery should not be blindly pursued for repair of hypospadias, especially in complicated and severe cases, which may increase the complexity of subsequent treatment. A range of staging surgical methods to repair hypospadias now exist. Active selection of the appropriate method is a technological and conceptual advancement (27, 28). This study compares the incidence of postoperative complications of the two staging surgical methods mentioned above, with an aim to determine their advantages and disadvantages.

In this study, the two types of staging surgical methods, i.e., prefabricated urethra and pre-implanted urethral plate, were used to repair severe hypospadias. Both of these were found to have lower rates of complications than that of one-stage surgery reported in a previous study (22), and the penis appearance was better than aero cyst that following single-stage surgery. Therefore, we believe that two-stage surgical repair could be adopted to treat severe hypospadias. However, the author proposes a comparative study of these two staging surgical methods so that a more suitable multi-staged procedure could be selected. The two types of surgery have different principles. The first procedure aimed to form a neo-urethra, then perform a strategic fistula exclusion to form the urethra in the first surgery and then to repair the fistula in the second stage. The second procedure aimed to reconstruct the urethral plate in the first stage, and to form the urethra in the second stage. Compared with the traditional one-stage plastic surgery method, the planned prefabricated urethra staging surgery has the advantage of avoiding the impregnation of the formed urethra with urine and urethral secretions, and allows full drainage of the urine, thereby reducing the pressure in the formed urethra. As a result, the occurrence of urinary fistula and urethrocele are reduced. The disadvantage is the heavier economic burden caused by multiple surgeries. Considering the overall outcome of reduced complications and the long-term effect, most doctors and patients accept the staging surgical procedure.

The pre-implanted urethral plate method includes two types, i.e., Byars and Bracka. Byars has the advantage of good blood supply to the skin flap and a small chance of necrosis; it also has the disadvantages of unsmooth transferred foreskin, which would cause urinary abnormality in the long run and difficulty in the formation of the glans in the second stage and the urethral orifice is often located in the coronary sulcus. Bracka has the advantages of a smooth urethra surface, which helps achieve unobstructed urination in the long run and satisfactory formation of the urethral orifice and the glans; the disadvantage is that if necrosis occurs to the freed implant, it will be difficult to repair with another surgery. The pre-implanted urethral plate method in this study adopted the Bracka method.

In clinical practice, the author found that the planned prefabricated urethra staging surgery method can reduce the occurrence of postoperative complications (such as urinary fistula, urethrostenosis, and urethrocele). However, the following problems also exist with this method: (1). In the case of glans hypogenesis (diameter < 1.4 cm), during reconstruction of the urethra, after the two sides of the glans were cut open, there was significant anastomosis tension at the glans, as a result of which it was prone to dehiscence. In this case, urethra reconstruction was changed to the tunnel method, which would increase the likelihood of external urethral orifice stenosis. (2). In cases where the foreskin blood vessels were in the form of a web without a main stem, the free pedicled skin flap will have poor blood supply during the formation of the urethra, resulting in a high likelihood of postsurgical urinary fistula and urethrostenosis. (3). The prefabricated urethra is a pedicled flap, in which local tissues are prone to edema, and the appearance of the penis is bloated and unsatisfactory.

The planned pre-implanted urethral plate staging surgery method has the following advantages: (1). In case of glans hypogenesis, the free skin flap is filled into the fully dissected glans wings during the first-stage surgery to increase the volume of the glans and reduce the tension of glans anastomosis during the second stage, hence preventing glans penis dehiscence and external urethral orifice stenosis. (2). The pre-implanted material applies free foreskin inner plate. The tissue is composed of abundant vasoganglia and fibroblasts, which has little subcutaneous fat tissue, no hair follicles, and strong ductility. This makes it easy to survive after the preimplantation, and this tissue is not prone to infection, necrosis, or lithiasis, and is resistant to urine stimulation (29). *In-situ* urethral anastomosis is performed in the second stage, without circular anastomotic stoma, and patients undergoing this surgery are not prone to urethrostenosis. In addition, the use of pedicled periorchium to strengthen coverage can reduce the occurrence of urinary fistula, while removing excess foreskin and subcutaneous tissue, preventing the penis from appearing bloated. (3). Circular or elliptical anastomosis when forming the external urethral orifice should be avoided. A “V-shaped” anastomosis can better contribute to the appearance of a slit-like external urethral orifice. (4). In the case of severe chordee and insufficient foreskin, joint transplantation of foreskin and labial mucosa can be used to reconstruct the urethral plate to provide material for the second-stage urethra formation. For pre-implanted urethral plate, some scholars have proposed that due to the lack of reliable blood supply, the use of free implant can easily cause ischemia and necrosis of the implants after the surgery. Therefore, the safety is not as high as compared with the prefabricated urethra (30). However, hyperbaric oxygen therapy is currently a recognized method to treat implants. Implants receiving hyperbaric oxygen therapy are healthier, and the area contracture rate and implant failure rate of these implants are significantly lower than those that do not receive hyperbaric oxygen therapy (31, 32). The author believes that if the implant is handled properly, and the hematocele between the implant and the penile body can be avoided to achieve better attachment, with postoperative hyperbaric oxygen therapy, the occurrence of hematocele is extremely small. Therefore, pre-implanted urethral plate can be used to plan the staging surgery for severe hypospadias, and the operation result is good. It is especially suitable for cases of glans hypogenesis, the absence of a main blood vessel in the foreskin, severe chordee, and insufficient foreskin (33).

PPPS (pediatric penile perception score) is a reliable instrument for assessing the postoperative cosmetic outcomes after hypospadias repair (34). According to our study, parents were less satisfied with the glans in group A. We suspect that because two cases of glans dehiscence were all in group A. We can also conclude that pre-implanted urethral plate method can achieve better penile appearance than prefabricated urethra method because both operators and counterparts were more satisfied in all items in group B.

This study has some limitations. First, it has a relatively small sample size as only 53 patients underwent staging urethroplasty. This was a single-center study, which may lead to introduction of selection bias for severe hypospadias patients, which may not be applicable to other clinic types. Additionally, the average follow-up duration was only 28 months. Patients with childhood repairs performed by pediatric urologists were often lost to follow-up during adolescence. Future multicenter studies with a longer follow-up and a larger sample size can help further verify these findings.

In summary, both the prefabricated urethra and the pre-implanted urethral plate had comparable results when used in the repair of severe hypospadias. However, the pre-implanted urethral plate staging operation had a wider range of indications; furthermore, the postoperative appearance of the penis and urethral orifice shape were more satisfactory with this procedure. Further research investigating these two approaches can further verify the findings of our study.

## Data Availability Statement

The original contributions presented in the study are included in the article/supplementary material, further inquiries can be directed to the corresponding authors.

## Ethics Statement

The studies involving human participants were reviewed and approved by The Ethics Committee of First People's Hospital of Zunyi City (Approval number: 2019-103). Written informed consent to participate in this study was provided by the participants' legal guardian/next of kin.

## Author Contributions

YD and SG conceived and designed research and conducted experiments. XX and ZY analyzed data. YD wrote the manuscript. All authors read and approved the manuscript.

## Funding

This work was supported by Zunyi science and technology program HZ(2019)190.

## Conflict of Interest

The authors declare that the research was conducted in the absence of any commercial or financial relationships that could be construed as a potential conflict of interest.

## Publisher's Note

All claims expressed in this article are solely those of the authors and do not necessarily represent those of their affiliated organizations, or those of the publisher, the editors and the reviewers. Any product that may be evaluated in this article, or claim that may be made by its manufacturer, is not guaranteed or endorsed by the publisher.
